# Quantification of blood flow in the portal circulation before and after an intervention

**DOI:** 10.1186/1532-429X-14-S1-W11

**Published:** 2012-02-01

**Authors:** Alejandro Roldán-Alzate, Alex Frydrychowicz, Scott B Reeder, Oliver Wieben

**Affiliations:** 1 Radiology, University of Wisconsin, Madison, WI, USA; 2 Medical Physics, University of Wisconsin, Madison, WI., USA

## Summary

PC-VIPR allows for characterization and quantification of hemodynamics in the entire hepatic system as well as inherent anatomical co-registration from a single scan. Results shown here reveal the potential of PC-VIPR for monitoring treatments for portal hypertension such as β-blockers and TIPS.

## Background

Hemodynamic and morphological assessment of portal hypertension (PHTN) is challenging due to the dual blood supply to the liver and complex and variable anatomy. Additionally, in the presence of liver disease such as cirrhosis, the increased resistance to flow leads to changes in hepatic and splanchnic blood flow, which if measured, could have diagnostic value. Accurate non-invasive assessment of hemodynamics and morphology is challenging yet has potential to improve our understanding and diagnosis of PHTN. The purpose of this study was to evaluate a volumetric 4D flow-sensitive MR imaging method using radial undersampling (PC-VIPR) for the analysis of portal circulation at baseline and after meal intervention.

## Methods

Six volunteers without history of liver disease were included in this IRB-approved study. PC-VIPR^1^ was performed on a 3T MR scanner before and after a meal intervention with: FOV=32x32x22cm, isotropic 1.3mm spatial resolution, TR/TE=6.1-7.8/2.1-3.2ms, Venc=60cm/s, scan time: ~10min using respiratory and retrospective ECG gating. Vessel segmentation was performed in MIMICs using PC angiograms. Cut-planes were placed in EnSight and analyzed in MatLab^2^. Flow (Q[ml/cycle]) in the superior mesenteric vein (SMV) and artery (SMA), splenic vein (SV), porta vein (PV) and hepatic artery (HA) was quantified. The imaging exam was performed after at least 5 hours of fasting. A meal consisting of 591mL EnSure plus® (Abbott Laboratories, Columbus, OH; 700cal, 28% from fat, 57% from carbohydrates) was provided to the subjects after a baseline scan. Scanning was resumed 20min after the meal. For internal validation, mass conservation at the splenomesenteric confluence was performed (Q_PV_=Q_SMV_+Q_SV_).

## Results

Segmentation quality of the hepatic and splanchnic angiograms was very good with excellent vessel detail in all cases. As shown in figure 2, large increases in flow were seen in the PV, SMV and SMA. Also, as expected, decreases in flow were seen in the SV and HA, although these decreases were not statistically significant. Finally, Internal validation (Q_PV_=Q_SMV_+Q_SV_) showed an acceptable error (5.9±3.4% and 6.9±5.5%, p=0.7) in the flow measurements at the confluence for baseline and intervention respectively.

## Conclusions

**Figure 1:**
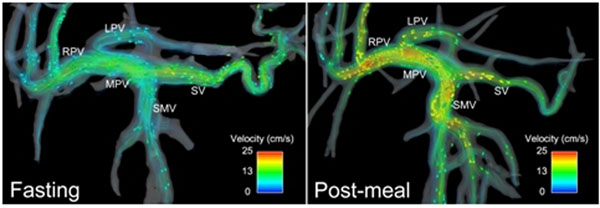
Changes in flow after a meal challenge in normal subjects (N=6). Increased flow is easily visualized in the portal and splanchnic circulation before (left) and after (right) a meal.

**Figure 2:**
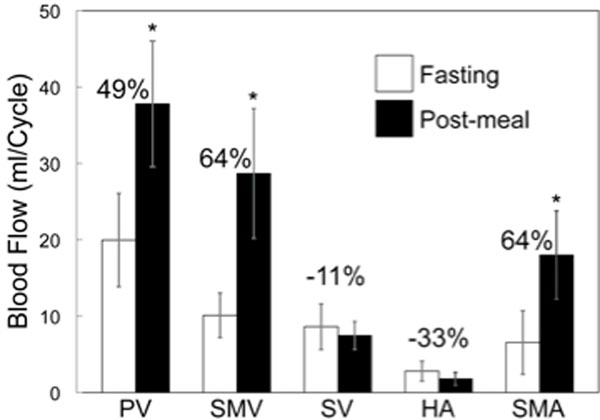
Flow measurement (right) were obtained via cut planes in vessels of interest. Flow increases in the PV, SMV and SMA, and decreases in the SV and HA as expected.

PC-VIPR allows for characterization and quantification of hemodynamics in the entire hepatic system as well as inherent anatomical co-registration from a single scan. Figures 1 and 2 show the hemodynamic changes in portal circulation induced by the meal challenge and demonstrate that PC-VIPR successfully quantify changes in flow. Internal validation results demonstrate the validity of hepatic blood-flow measurements with PC-VIPR. These results show the potential of PC-VIPR for monitoring treatments for portal hypertension such as β-blockers and TIPS.

## Funding

University of Wisconsin - Madison, Department of Radiology, Research and Development fund. R01 DK083380, R01 DK088925, RC1 EB010384.

## References

[B1] Johnson K MRM 2010

[B2] Stalder A MRM 2008

